# N- and S-doped high surface area carbon derived from soya chunks as scalable and efficient electrocatalysts for oxygen reduction

**DOI:** 10.1088/1468-6996/16/1/014803

**Published:** 2015-02-18

**Authors:** Moumita Rana, Gunjan Arora, Ujjal K Gautam

**Affiliations:** 1 New Chemistry Unit, Jawaharlal Nehru Centre for Advanced Scientific Research, Bangalore 560064, India; 2 Department of Chemistry, Hansraj College, University of Delhi, Delhi 110007, India

**Keywords:** N-doped carbon, fuel cell, oxygen reduction reaction

## Abstract

Highly stable, cost-effective electrocatalysts facilitating oxygen reduction are crucial for the commercialization of membrane-based fuel cell and battery technologies. Herein, we demonstrate that protein-rich soya chunks with a high content of N, S and P atoms are an excellent precursor for heteroatom-doped highly graphitized carbon materials. The materials are nanoporous, with a surface area exceeding 1000 m^2^ g^−1^, and they are tunable in doping quantities. These materials exhibit highly efficient catalytic performance toward oxygen reduction reaction (ORR) with an onset potential of −0.045 V and a half-wave potential of −0.211 V (versus a saturated calomel electrode) in a basic medium, which is comparable to commercial Pt catalysts and is better than other recently developed metal-free carbon-based catalysts. These exhibit complete methanol tolerance and a performance degradation of merely ∼5% as compared to ∼14% for a commercial Pt/C catalyst after continuous use for 3000 s at the highest reduction current. We found that the fraction of graphitic N increases at a higher graphitization temperature, leading to the near complete reduction of oxygen. It is believed that due to the easy availability of the precursor and the possibility of genetic engineering to homogeneously control the heteroatom distribution, the synthetic strategy is easily scalable, with further improvement in performance.

## 1. Introduction

Increasing global energy demand and the continued depletion of fossil fuel have led to the development of a number of highly promising alternative energy strategies. Among them, fuel cells are important due to the potential generation of very high power density and green operating conditions. Although the performance of a polymer electrolyte membrane fuel cell (PEMFC) has improved in recent times, it is of paramount importance to develop better catalysts to counter the sluggish kinetics of an oxygen reduction reaction at the cathode [1]. Usually, noble-metal-based electrocatalysts are considered to be the best catalyst for an oxygen reduction reaction (ORR) in fuel cells [2–5]. However, high cost and lack of abundance limit their utilization [6]. Furthermore, these materials also suffer from continuous degradation in performance due to surface-poisoning, such as CO deactivation and alcohol cross-over, and often have short-term stability in electrochemically harsh conditions [7–9]. On the other hand, non-metallic catalysts, such as carbon materials, do not have such disadvantages, and large efforts have recently been made to match their performance with Pt-based catalysts [10]. It is found that the doping of heteroatoms, such as nitrogen, phosphorus, sulphur and boron into the carbon skeleton [11–13], assist oxygen adsorption and reduction by changing the polarity of neighbouring carbon atoms, thereby enhancing ORR activity [14]. From theoretical studies it has been found that in doped carbons, the catalytically active sites are the carbon atoms next to heteroatoms, which show excellent bonding ability to adsorb OOH, thus aiding the easy formation of H_2_O_2_, which upgrades their catalytic activity [15].

Different chemical strategies were used for the synthesis of doped and high surface area porous carbon; [16, 17] these are based on treatment with a precursor of a heteroatom in pure carbon-based materials such as graphene [18, 19] and carbon nanotubes [20]. Such chemical processes involve multiple processing steps, are relatively expensive and are difficult to scale up for industrial applications. Moreover, these approaches may not lead to the homogeneous doping of heteroatoms in a carbon matrix [21]. On the other hand, the synthesis of such a doped carbon network could be much easier when the synthesis is performed using a precursor that has an inbuilt homogeneous source of heteroatoms. Using this concept, N-doped carbon materials have been synthesized from egg protein and other animal sources [22–27]. Chaudhari *et al* have shown that using human hair, an efficient carbon catalyst was obtained, which was simultaneously doped with two heteroatoms: nitrogen and sulphur [28]. Recently, it was found that phosphorus co-doping of a carbon network can further promote ORR activity [29–31]. Based on these observations, one may envision that a plant source for obtaining co-doped carbon catalysts would be highly advantageous as they are easily grown in large quantities and can be genetically modified to control the doping contents [32, 33]. However, to the best of our knowledge, there is no such study for co-doping of nitrogen, sulphur and phosphorus all together in one material using biomass as a precursor.

**Figure 1.**
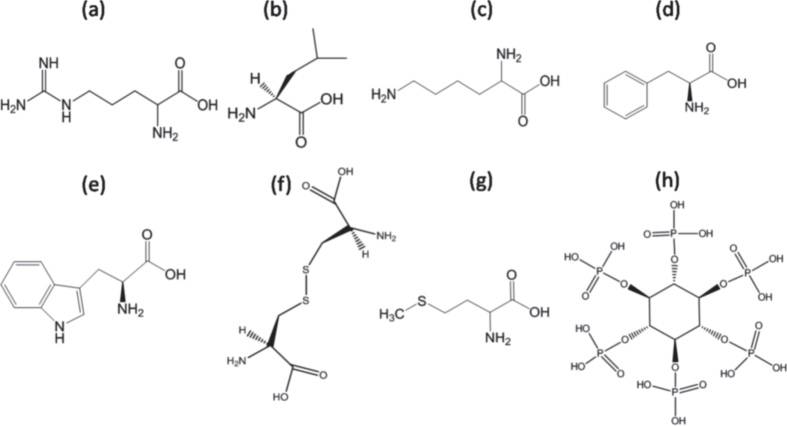
Structure of molecules as heteroatoms sources in *glycine max*: (a) arginine, (b) leucine, (c) lysine, (d) phenylalanine and (e) tryptophan as N sources; (f) cysteine and (g) methionine as S sources; and (h) phytic acid as the P source.

Soya (*glycine max*) is used as a food ingredient and is widely available as a less-expensive processed food with a high protein content (>50%, carbohydrate content ∼30%). Its constituent molecules, such as cystine and methionine, contain sulphur, while lycine, threonine, leucine, isoleucine, valine, tryptophan, phenylalanine and arginine contain nitrogen bonded to a carbon atom (figure 1). It is also rich in phytic acid with a high phosphorus content. Therefore, we presumed that pyrolysis of soya chunks would lead to a conducting carbon network doped with essential heteroatoms. In this manuscript, we show that soya is a natural precursor to obtain large quantities of an excellent low-cost carbon-based catalyst with a high surface area (∼1000 m^2^ g^−1^) for use in a fuel cell cathode. During ORR measurements in basic media, an onset potential of −0.045 V as well as high efficiency with a ∼4 electron reduction pathway was recorded, which is comparable to that of commercial Pt catalysts. It has better long-term stability compared to commercial Pt. Furthermore, it exhibited excellent methanol tolerance, unlike the metal-based catalysts, demonstrating a promising alternative for costly Pt-based electrocatalysts.

## 2. Experimental section

### 2.1. Chemicals

The glycine (soya) chunks (brought from a supermarket), NaOH (98.0%, SDFCL) and HCl (35.4%, SDFCL) were used as received. The aqueous solutions were prepared using ultrapure water (>18.2 M*Ω* cm, Milli-Q Plus system (Millipore).

### 2.2. Syntheses

**Figure 2.**
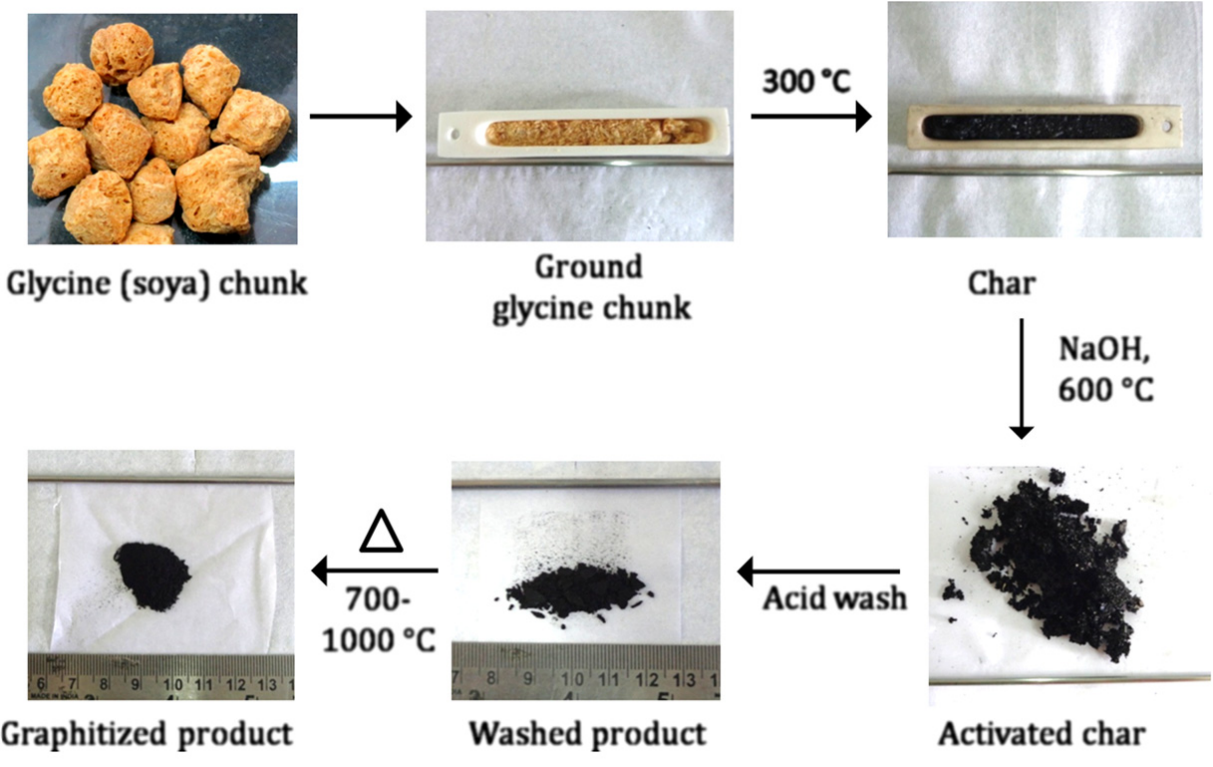
Outline of the synthesis procedure adopted for generating high surface area heteroatom-doped carbon from a glycine chunk.

The conversion of soya to an efficient ORR catalyst involves multiple steps, which can be broadly divided into three parts, as described below. The process is summarized in figure 2.

#### 2.2.1. Pre-carbonization

Soya chunks (SC) were powdered and transferred to an alumina boat. It was placed at the centre of the quartz tube furnace (Elite THS/15/50/180-2416 CG). Ar was allowed to flow for half an hour before increasing the temperature in order to maintain an inert atmosphere, and the furnace was heated to 300 °C for 3 h to produce a partially carbonized substance, mentioned as char hereafter.

#### 2.2.2. Activation with NaOH

Char was ground into a fine powder and mixed with NaOH in a 1:3 ratio (char: NaOH, wt/wt) for the activation. The obtained mixture was then transferred to an alumina crucible and placed at the centre of a tube furnace in an Ar atmosphere. Activation was done at 600 °C with a heating rate of 10 °C min^−1^ and holding time of 1 h under a continuous Ar flow. The obtained product was allowed to cool naturally and treated with 1.0 M HCl and deionized water to neutralize excess NaOH as well as other naturally occurring metal precursors. It was washed several times with deionized water, followed by ethanol, and dried in a vacuum oven maintained at 70 °C. A large amount of this sample was collected by executing the same approach. This activated carbon (SC-600) was used as the precursor material for further experimentation.

#### 2.2.3. Graphitization

The dried SC-600 was graphitized at different temperatures ranging from 700 °C to 1000 °C in an Ar atmosphere for two hours. The obtained samples were thus named as SC-T, where T stands for graphitization temperature.

The complete synthesis procedure for obtaining the graphitized carbon from a pristine soya chunk is shown in figure 2.

### 2.3. Structure characterization

The structural features of the as-prepared samples were investigated by scanning electron microscopy (SEM) using a FEI Quanta 3D FEG instrument. Energy-dispersive x-ray spectroscopy (EDAX) analysis was performed on an Apollo XL instrument fitted with a Quanta 3D-5 Microscope (Port: EDAX). Transmission electron microscopy (TEM) was performed using a JEOL, JEM 3010 instrument (300 kV) fitted with a GATAN CCD camera. The powder x-ray diffraction (PXRD) patterns were recorded on a Bruker AXS, D8 Discover x-ray diffractometer using Cu K*α* irradiation (*λ* = 1.54187 Å) in a 2*θ* range from 15 to 90°. A TGA 850C, Mettler Toledo thermogravimetric analyzer was used to evaluate the weight loss on heating. 10 mg of the sample was heat-treated in a ceramic crucible from 30 to 900 °C at a constant heating rate of 5 °C min^−1^ under a N_2_ atmosphere (flow rate of 40 mL min^−1^). To study the surface properties, x-ray photoelectron spectroscopy (XPS, VG Scientific ESCA LAB V) was used. Raman spectroscopy was performed at different locations of the sample using a Jobin Yvon LabRam HR spectrometer with a 514.5 nm Ar laser. N_2_ and O_2_ adsorption studies were performed at a 77 K temperature using a QUANTACHROME QUADRASORB SI analyzer and an AUTOSORB IQ2 instrument. Prior to the adsorption studies, all the samples were kept at 200 °C under high vacuum for 12 h.

### 2.4. Electrode preparation and electrochemical measurements

For evaluating the electrochemical performances of the final pyrolized samples, a rotating disk electrode (RDE) technique was used. A mixture of a 3 mg sample, 0.5 mL Millipore water, 0.5 mL ethanol and 25 *μ*L nafion (5%) was sonicated for half an hour and used as catalyst ink. 10 *μ*L of this catalyst ink was drop-cast on a glassy carbon electrode. The electrode was dried overnight in an ambient atmosphere. A 0.1 mol L^−1^ KOH solution was used as an electrolyte. The counter and reference electrodes were a platinum coil and a saturated calomel electrode (SCE), respectively.

All the electrochemical measurements were recorded on an electrochemical workstation (CHI760E and RRDE-3A). Linear sweep voltammetry (LSV) measurements were recorded at rotating speeds of 400, 600, 900, 1200, 1600 and 2000 rpm in an O_2_-saturated electrolyte at a sweep rate of 5 mV s^−1^. The electrode was then scanned in an Ar-saturated electrolyte in similar conditions to determine the background current. The number of electrons involved in the reduction process was calculated by the Koutecký–Levich (K–L) equation.1
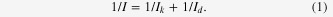
Here *I, I*_*k*_ and *I*_*d*_ are the total measured current, kinetic current and diffusion limited current respectively. The dependence of *I*_*d*_ on the rotation speed can be expressed as2
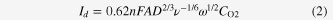
where *n* is the number of electrons involved in the reaction, *F* is Faraday’s constant (96 485 C mol^−1^), *A* is the area of the electrode (0.0707 cm^2^), *D* is the diffusion coefficient of O_2_ in the electrolyte (1.93 × 10^−5^ cm^2^ s^−1^), *ν* is the kinematic viscosity of the electrolyte (1.01 × 10^−2^ cm^2^ s^−1^), *ω* is the angular frequency in the rpm of the RDE, *C*_O2_ is the concentration of O_2_ in the electrolyte (1.26 × 10^−6^ mol cm^−3^).

The stability of the samples was checked by performing chronoamperometry at a constant voltage of −0.4 V and at an electrode rotation rate of 1600 rpm. The methanol tolerance of the samples was obtained by chronoamperometry by adding 4 mL methanol at 200 s.

## 3. Result and discussion

**Figure 3.**
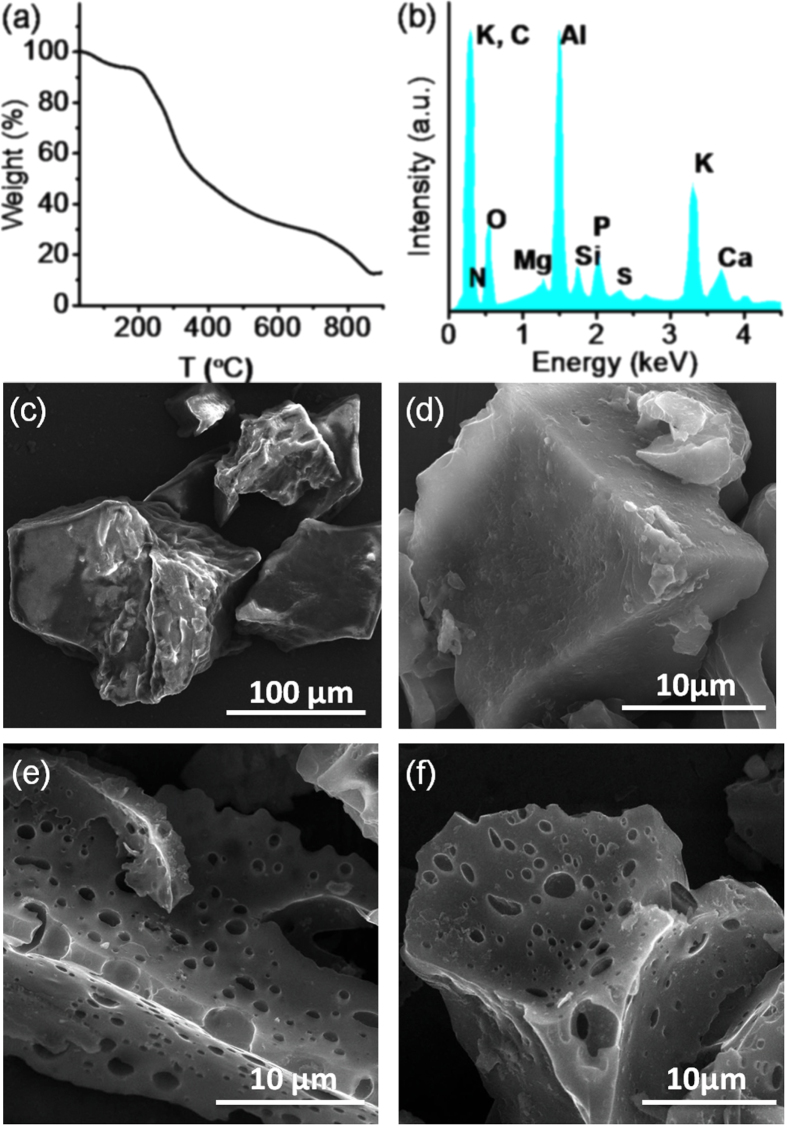
(a) TGA plot of a glycine chunk in an Ar atmosphere. (b) EDAX spectrum of char pyrolyzed at 300 °C showing the presence of O, N, S and P. SEM images of (c) a glycine chunk, (d) char and (e), (f) NaOH activated carbon, SC-600.

During the pyrolysis of the soya chunk at 300 °C, the oil content of the soya first evaporated and was deposited as reddish-yellow slurry on the furnace tube, leaving behind primarily the protein and carbohydrate part. Thermogravimetric analysis (TGA, figure 3(a)) of the soya chunks exhibited a continuous weight loss of ∼68% with a sharp drop around 300 °C, which can be attributed to the removal of oils and other small molecules. The char obtained at 300 °C maintained a chunk-like morphology almost without any noticeable features such as surface-porosity (figures 3(c) and (d)). In order to instill pore-like features to increase the surface area, activation of this char was carried out at a high temperature (at 600 °C) by NaOH etching. During etching, the NaOH reacts with the carbon precursors, leading to the development of functional groups such as –ONa. Due to the formation of such –ONa bonds in char, oxidation of the cross-linking carbon atoms in the adjoining lamella takes place, resulting in the rupture of cross-linking between neighbouring lamella. As the lamellae are disturbed from their usual configuration into a slightly wrinkled form, they are unable to regain their original nonporous state upon cooling, leading to interlayer voids and producing porosity and high surface area carbon [34, 35]. In figures 3(e) and (f), we show SEM images of the etched materials, clearly demonstrating porous material and the effect of chemical etching. However, no transparent sheet-like features observed for graphene-based materials were observed at this stage of treatment.

**Figure 4.**
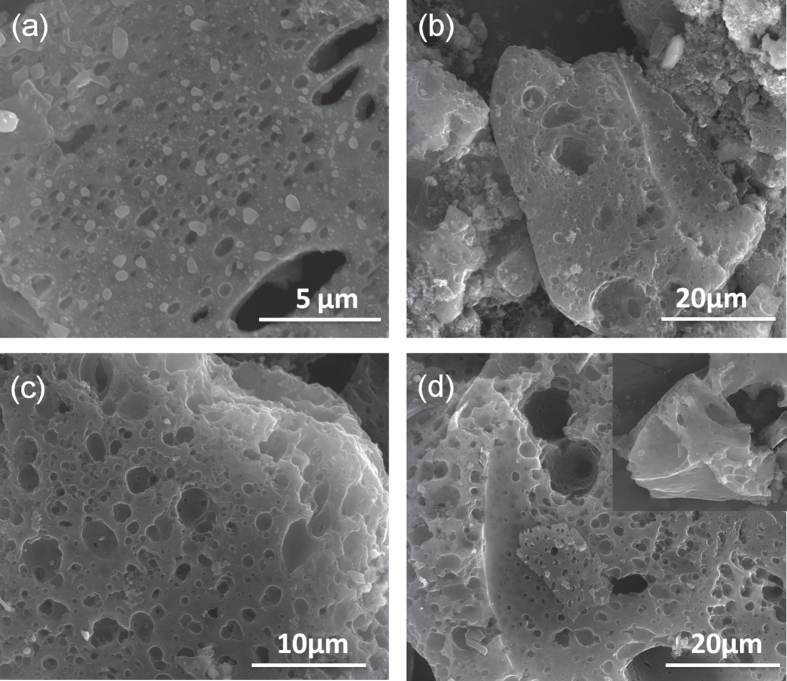
SEM images of (a) SC-700, (b) SC-800, (c) SC-900 and (d) SC-1000 showing the formation of porous wrinkled surfaces during high temperature treatment.

**Figure 5.**
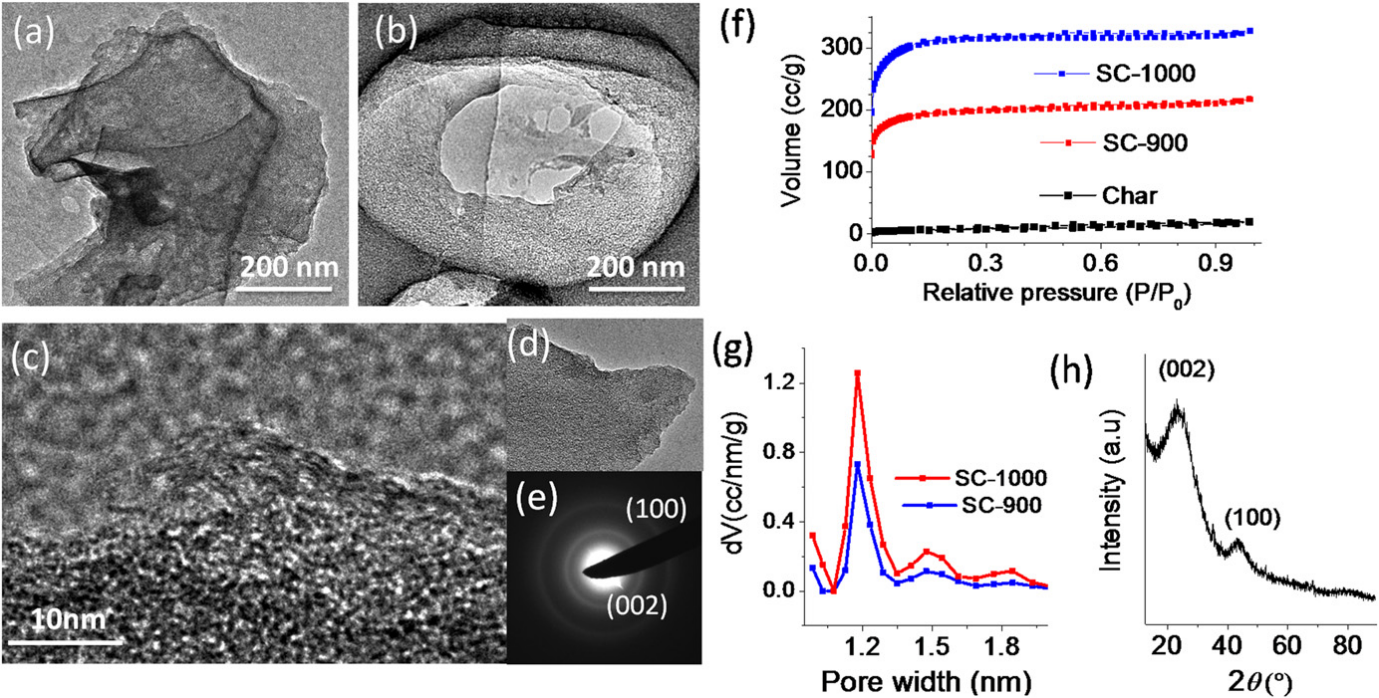
(a), (b), (d) TEM images of SC-1000 showing the formation of a transparent sheet-like morphology. (c) TEM image of an edge showing graphene-like features. (e) SAED on doped carbon showing the diffused ring patterns generated from a graphene-like region. (f) N_2_ adsorption and desorption profile of char, SC-900 and SC-1000. (g) NLDFT pore size distribution of SC-900 and SC-1000. The narrow peaks centred at ∼1.2 nm correspond to uniform nanopores present in both samples. (h) PXRD pattern of SC-1000.

The SC-600 was further graphitized at higher temperatures (700–1000 °C) to obtain heteroatom high surface area doped carbon-based materials. Depending on the temperature of the graphitization, the yield of the material was found to vary between 55–70 weight %. The microscopic features of the graphitized samples are shown in figure 4, which contained porous wrinkled structures that resemble the porous features of the etched materials. However, careful observation of the images infers that an increase in the graphitization degree leads to an increase in the surface roughness and porosity of the material. The sample obtained at 1000 °C is highly wrinkled and contains sheet-like structures too. TEM investigations on the SC-1000 (figures 5(a)–(d)) showed a transparent wrinkled sheet throughout the sample. Figure 5(c) shows few-layer-graphene-like features, usually observed for this sample at the pore edges. We also recorded selected area diffraction patterns (SAED) at various portions of the sample, which confirmed the formation of a graphitized region across the sample (figure 5(e)). The uneven, wrinkled surface structure of these materials compared to the SC-600 is generated by the evaporation of small molecules (including oxides and sulphides of carbon, as we observed a reduction of the O and S content after graphitization) at a high graphitization temperature. In addition, since the decrease of O, S and P content is higher (supported by XPS and Raman data, as described later) with the increasing process temperatures, we speculate that a higher temperature leads to more evaporation around the heteroatoms. This also possibly leads to higher surface area of the material.

Due to the porous appearance of the materials and the need for high surface area for efficient ORR, we subsequently analyzed the surface area of the important SC-900 and SC-1000 samples. A significant change in the Brunauer–Emmett–Teller (BET) surface area (s.a.) as well as an increase in the graphitization temperature (figure 5(f)) was observed upon activation. In comparison to the char (s.a. = ∼25 m^2^ g^−1^), SC-900 exhibited a high surface area of 607 m^2^ g^−1^. The s.a. further increased to 1072 m^2^ g^−1^ when the graphitization temperature was 1000 °C, which is much higher than that of commercial electrocatalyst carbon support (vulcan XC 72, ∼600 m^2^ g^−1^) and comparable to many stateof-the-art carbon-based catalysts (as shown in table 2). Using the nonlocal density functional theory (NLDFT) calculation to analyze the adsorption isotherm, we have observed the presence of pores in these samples with a narrow diameter distribution (1.2 nm for both samples). The calculated pore volumes using N_2_ as a probe (kinetic diameter ∼3.64 Å) in the NLDFT method were found to be 0.303 and 0.455 cm^3^ g^−1^ for SC-900 and SC-1000, respectively. The PXRD pattern of SC-1000 shows (figure 5(h)) two broad peaks centred at 23.8° and 43.6° that correspond to the (002) and (100) lattice planes of graphitized carbon, respectively [36].

**Figure 6.**
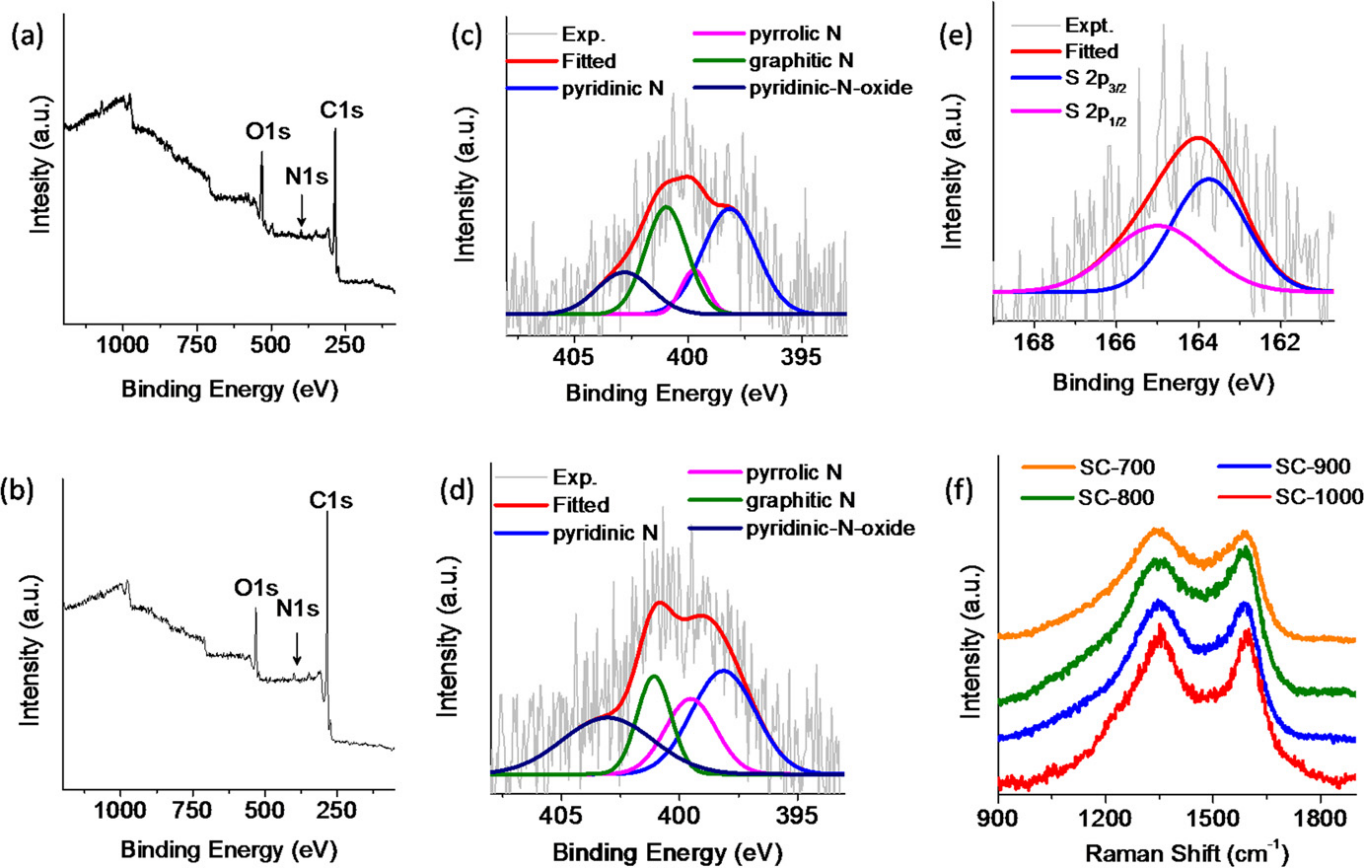
XPS spectra of (a) SC-900 and (b) SC-1000. High-resolution XPS spectra for the N 1s transition of (c) SC-900 and (d) SC-1000. (e) High-resolution XPS spectrum for the S 2p transition of SC-900. (f) Raman spectra of the graphitized samples.

The surface compositions of SC-900 and SC-1000 were analyzed by XPS. As anticipated, on the basis of the composition of the soya precursor, in the survey spectra both the samples show the predominant presence of carbon (1s, 284.6 eV) along with heteroatoms O (1s, 532 eV) and N (1s, 399.5 eV) in them (figures 6(a) and (b)) [37]. High-resolution XPS spectra were further collected to quantify the doping amounts of the heteroatoms in the carbon framework (figures 6(c)–(e)). The relative ratios of atoms are tabulated in table 1. It revealed that as the graphitization temperature increases, the oxygen quantity gradually decreases. On the other hand, the N content remains unaltered at this temperature range. In both cases the N-1s peak can be deconvoluted into four peaks: pyridinic-N (398.15 eV ± 0.2), pyrrolic –N (399.55 eV ± 0.2), graphitic-N (401.1 eV ± 0.2) and pyridinic oxide-N (403.3 eV ± 0.2) [38]. The atomic percentages of each type of these N are 1.51, 0.9, 0.71 and 1.2% for SC-900 and 2.2, 0.47, 1.75 and 0.85% for SC-1000. Similarly, the S content also decreases with the increasing temperature, and no S was detected in the case of the SC-1000. The peak corresponding to sulphur (2p, 164.2 eV) was observed only in the case of the SC-900, which can be deconvoluted to two peaks corresponding to the 2p_1/2_ (163.9 eV) and 2p_3/2_ (164.8 eV) transitions [11]. However, notwithstanding our expectations of P-doping due to its presence in the precursor, we could not detect any signal pertaining to P in the XPS spectra. A small quantity of this was however observed very rarely in EDAX measurements. It is possible that P might have been removed in one of the processing steps as phosphate ions; another possibility is the evaporation of phytic acid (flash point 674 °C) at high reaction temperatures. Unlike O, S and N atoms, which are connected to a C atom in the precursor, P atoms are not directly connected to C and are instead connected to four oxygen atoms as a phosphate ion. This situation may not offer an opportunity for P to get incorporated into the carbon framework in our reaction condition, though phosphates are a preferred precursor for P-doping in carbon [29, 31].

**Table 1.** Key structural features and ORR activity parameters related to SC-900 and SC-1000.

Sample name	SC-900	SC-1000
Structural features	C-content	79.4%	84%
	O-content	15.5%	10.7%
	N-content	4.3%	5.3%
	S-content	0.8%	—
	Surface area	607 m^2^ g^−1^	1072 m^2^ g^−1^
			
ORR activity (versus SCE)	Onset potential	−0.091 V	−0.045 V
	Half-wave potential	−0.227 V	−0.211 V
	No. of e-transfer	3.1	3.7
	Current at −0.4 V	7.48 A g^−1^	8.81 A g^−1^
	Current loss after 3000 s	7.1%	5.5%

Further insights into the effect of doping and the nature of graphitization in the different carbon samples were obtained using Raman spectroscopy. Figure 6(f) shows the Raman spectra of SC-700, SC-800, SC-900 and SC-1000 depicting the characteristic D and G bands of graphitic carbon, which appear at 1350 cm^−1^ and 1580 cm^−1^, respectively. The G band is generated from a Raman active E_2g_ mode of the graphitic environment (sp^2^ carbon). The D band, on the other hand, is generated from the zone-edge A_1g_ mode, which appears due to the presence of disorder arising out of defects or heteroatoms (such as O, N, S, P) and unfavourable carbon hybridization (such as sp^3^ carbon) [39, 40]. A higher degree of disorder leads to a broader and more intense D band. As can be clearly observed from the spectra, disorder in the carbon framework is much higher when the graphitization temperature is low. This also indicates, in support of the XPS investigations, the decrease in the heteroatom content and a higher degree of graphitization at high treatment temperatures.

**Figure 7.**
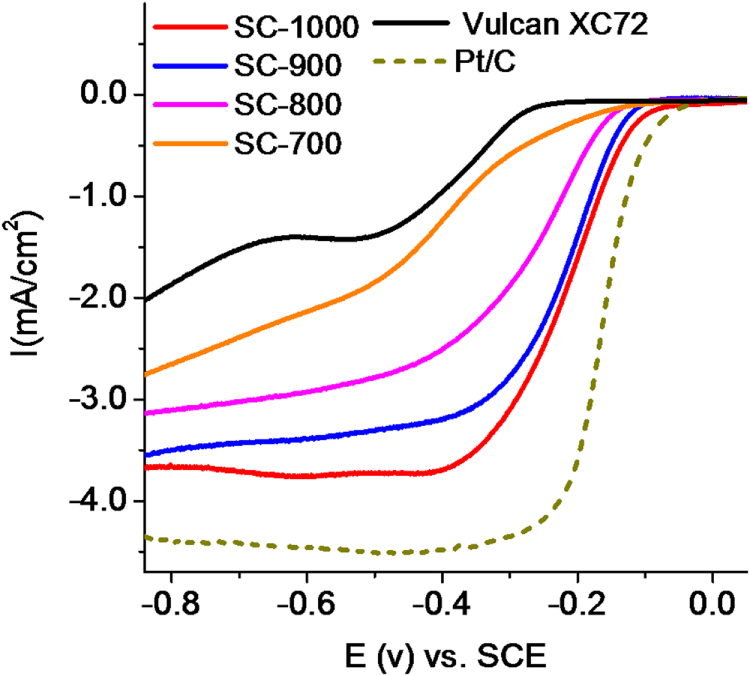
Linear sweep voltammograms (LSV) of different graphitized samples and their comparison with commercial Pt/C and amorphous carbon (Vulcan XC72) performed in an O_2_ saturated 0.1 M KOH solution at 1600 rpm and at a scan rate of 5 mV s^−1^.

Based on the above observations, we found that with the increasing processing temperature, graphitization of soya leads to the following: (1) an increase in the extent of graphitization, (2) an alteration of the extent of heteroatom doping, (3) an increase in surface area. It is possible that due to these factors, conductivity of the samples also changes with the processing temperature. Although it is difficult to envision the contribution of each factor toward electrocatalytic ORR activities of these materials, certainly some of them ought to outweigh the others in favour of higher efficiency. The electrocatalytic activities of these pyrolyzed samples were investigated for ORR by RDE using a three-electrode electrochemical system in an Ar- and O_2_-saturated 0.1 M KOH solution, respectively. In order to compare their performance with a standard catalyst, the performance of a commercially available Pt-loaded carbon was further estimated. Figure 7 shows the linear sweep voltammograms of all the samples in an O_2_-saturated 0.1 M KOH solution collected using a scan rate of 5 mV s^−1^ at 1600 rpm. It was found that the catalyst parameters steadily improve with the increased processing temperature. For SC-1000, we have recorded onset and half-wave (E_1/2_) potentials of −0.045 V and −0.211 V, respectively. The performance of SC-900 is somewhat poorer (onset −0.091 V and E_1/2_ = −0.227 V, respectively. In particular, the onset potentials of these two samples are comparable to that of C-Pt/C (onset −0.021 V, E_1/2_ = −0.172 V). SC-800 and SC-700 have shown comparatively suppressed ORR activity with onset and half-wave potentials of −0.129 V, −0.271 V and −0.144 V, −0.413 V, respectively. Although the onset potentials of SC-1000 and 900 are almost the same, the E_1/2_ as well as the saturation currents for SC-1000 are superior to SC-900. Considering the composition and structural features of both samples, we believe that the lower onset potential of SC-900 is due to the higher heteroatom content. On the other hand, a high pyrolysis temperature for SC-1000 causes better graphitization (as evident from Raman spectra) which in turn may increase the conductivity of the sample and consequently E_1/2_. The higher saturation current for SC-1000 is due to its higher surface area in comparison to SC-900. In order to evaluate the effect of heteroatom doping, we have also performed ORR using commercially available amorphous carbon, Vulcan XC72 (with a BET surface area of 265 m^2^ g^−1^), under identical conditions. As seen in figure 7, it has exhibited negligible ORR activity with an onset of −0.212 V and very little saturation current.

**Figure 8.**
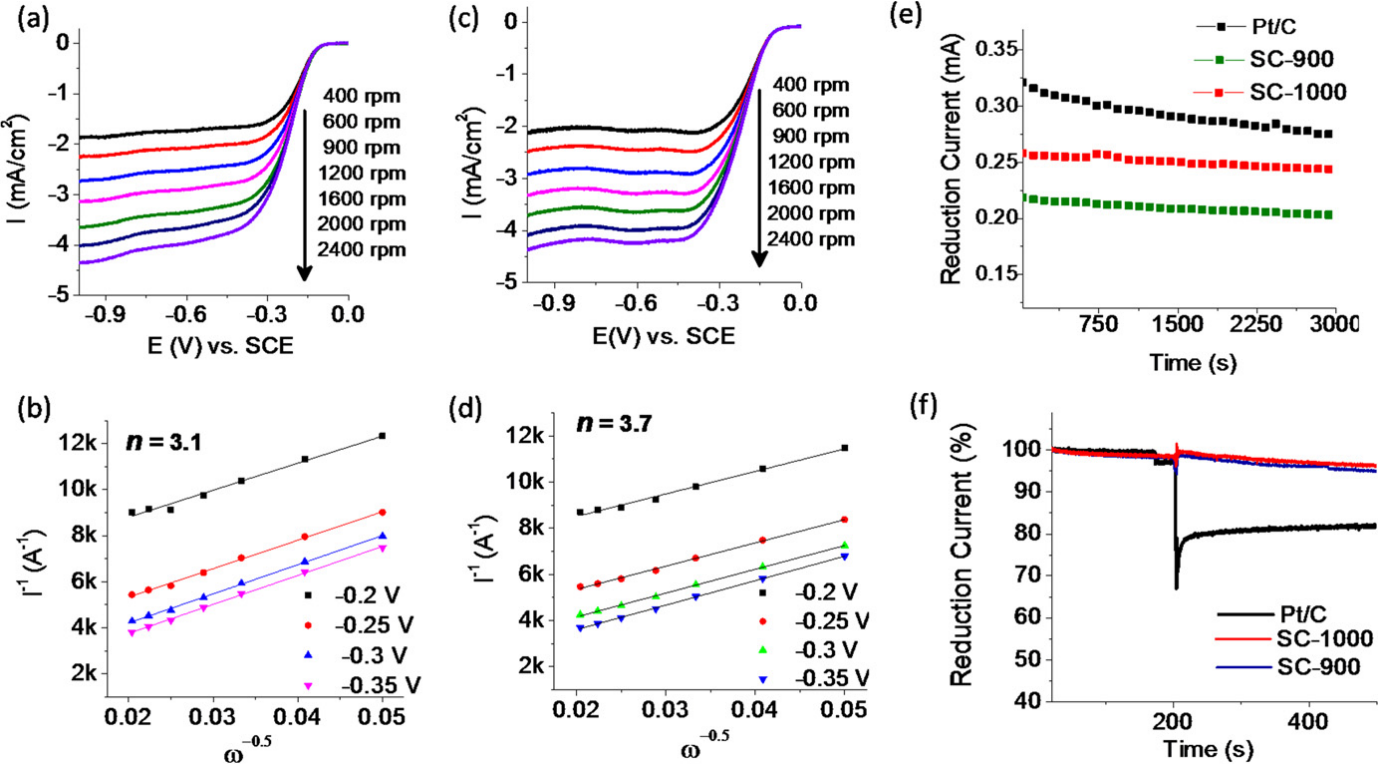
(a), (c) LSV plots obtained with different rotation rates in the range of 400–2000 rpm (scan rate 5 mV s^−1^) and (b), (d) the corresponding K–L plot for (a), (b) SC-900 and (c), (d) SC-1000. (e) Chronoamperomteric current-time (*I/t*) response of SC-900, SC-1000 and commercial Pt/C, performed at a voltage of −0.4 V (versus SCE) at 1600 rpm. (f) *I/t* chronoamperometric response of SC-900, SC-1000 and commercial Pt/C at −0.4 V, 1600 rpm for the estimation of methanol tolerance (added after 200 s). All the measurements were performed in an O_2_-saturated 0.1 M KOH solution.

The LSV for SC-1000 and SC-900 were collected at different rotation rates to estimate the number of electrons (***n***) transferred per oxygen molecule during the reduction of oxygen and the corresponding ORR efficiency by employing the Koutecky–Levich equation (figures 8(a)–(d)). ***n*** for SC-900 and SC-1000 were found to be 3.1 and 3.7, respectively, indicating nearly complete reduction of oxygen by SC-1000. On the other hand, we believe that a considerable amount of H_2_O_2_ is generated while using SC-900. It may be noted that it is the graphitic N in a carbon framework that favours the 4e- reduction process, while the pyridinic N leads to the 2e-reduction process, leading to H_2_O_2_ production [10, 37, 38]. From the XPS investigations of these samples, since SC-1000 contains a higher content of graphitic N (1.75 atomic % compared to 0.71% for SC-900), it has exhibited a higher number of electron transfers. Further, to check the stabilities of these samples, we performed chronoamperometry at a constant potential of −0.4 V, where the reduction current nearly saturates, for 3000 s at 1600 rpm (figure 8(e)). The decrease in the reduction currents were found to be 5.48, 7.1 and 13.9% for samples of SC-1000, 900 and commercial Pt/C, respectively. Finally, the methanol tolerance for our catalysts were checked, since methanol is a common catalyst-poison for noble metal catalysts in a direct methanol fuel cell. We added 4 mL of methanol in 80 mL of an electrolyte at 200 s while performing chronoamperometry at −0.4 V at 1600 rpm. As can be seen for our samples in figure 8(f), no significant change in reduction current was observed before and after the addition of methanol, whereas due to the oxidation of methanol on Pt/C, its catalytic activity decreased abruptly post methanol addition. Based on these performance parameters, including the onset and half-wave potential and the electron transfer number and stability, SC-1000 is the best performing catalyst among all our samples. We therefore compared its performance with other recently reported important carbon-based catalyst systems. As seen from table 2, SC-1000 represents one of the best carbon-based ORR catalysts reported with a very high onset and half-wave potential.

**Table 2.** Comparison of the performance of SC-100 toward ORR with other state-of-the-art carbon-based electrocatalysts.

S. No.	Material	Surface area (m^2^ g^−1^)	N content (atomic %)	Onset potential^a^ (V)	Half-wave potential^a^ (V)	References
1.	Soya-derived heteroatom-doped carbon	1062	5.3	0.96	0.79	*This work*
2.	N and S co-doped graphene	—	5	0.908	0.708	[41]
3.	N and P dual-doped porous carbon foams	755.7	3.7	0.947	0.777	[42]
4.	N-doped porous carbon nanopolyhedra	932	4.8	0.95	0.779	[43]
5.	N-doped carbon sheets derived from gelatin	933.9	1.41	0.95	0.75	[44]
6.	Co–N–C hybrid using soya milk	—	0.85	0.807	0.717	[33]
7.	N-doped graphene	—	4	0.822	0.672	[45]
8.	P-doped ordered mesoporous carbons	930	—	0.854	0.774	[46]
9.	N-doped graphene	—	8.3	0.842	0.632	[47]
10.	Nanoporous N-doped graphene	1000	4.9	0.892	0.672	[48]
11.	Chicken-bone-derived N-doped porous carbon	769	—	0.914	0.784	[49]
12.	Hair-derived N, S-doped carbon	1548.46	3.8	0.956	0.825	[28]
13.	N-doped multilayer graphene from milk powder with melamine	—	7.41	0.879	0.749	[21]
14.	N-doped carbon using pulse flour	750	1	0.949	0.7	[32]

a For easy comparison, we have converted the reported values of potentials with respect to a reversible hydrogen electrode (RHE) using the Nernst equation.

## 4. Conclusions

In conclusion, we have developed a highly efficient electrocatalyst for oxygen reduction reaction using soya as a natural precursor for doped and high surface area carbon. Being rich in protein, soya acts as a simple, cultivable and easily available source of high-quantity nitrogen. It has been found that the doping concentration and the degree of graphitization can be tailored by choosing the appropriate processing parameters. Moreover, the surface areas of these materials are much higher than those of commercially available carbon materials; their surface area is dependent on the treatment temperature. The overall catalytic performance of these materials is expected to be controlled by the contribution of all these factors, and the sample prepared at 1000 °C offers the best performance toward ORR, which is comparable to a commercially available Pt catalyst. On the other hand, the efficiency of this catalyst is far superior to commercial carbon. We believe that our method is easily scalable, and since processed soya chunks easily absorb a variety of solvents and solutions, our method can also be extended to obtain other catalyst systems such as metals or metal-oxide-based catalysts.
